# Frailty is an independent risk factor for recurrence and mortality following curative resection of stage I–III colorectal cancer

**DOI:** 10.1002/ags3.12337

**Published:** 2020-04-19

**Authors:** Kosuke Mima, Nobutomo Miyanari, Atsushi Morito, Shinsei Yumoto, Takashi Matsumoto, Keisuke Kosumi, Mitsuhiro Inoue, Takao Mizumoto, Tatsuo Kubota, Hideo Baba

**Affiliations:** ^1^ Department of Surgery National Hospital Organization Kumamoto Medical Center Kumamoto Japan; ^2^ Department of Gastroenterological Surgery Graduate School of Medical Science Kumamoto University Kumamoto Japan

**Keywords:** elderly, geriatrics, gerontology, morbidity, neoplasm

## Abstract

**Aim:**

With population aging, the number of frail patients with colorectal cancer has increased. The Clinical Frailty Scale (CFS) is a validated tool for assessing frailty, and higher scores indicate worse clinical outcomes following cardiovascular procedures. This retrospective study aimed to examine preoperative frailty in relation to recurrence and mortality following curative resection of colorectal cancer.

**Methods:**

We retrospectively analyzed data for 729 consecutive patients undergoing curative resection of stage I–stage III colon and rectal adenocarcinoma between January 2009 and December 2016. Frailty was assessed using the CFS: 1 (very fit) to 9 (terminally ill), and frailty was defined as CFS ≥ 4. Recurrence‐free survival (RFS) and overall survival (OS) were compared between frail and nonfrail patients. Cox proportional hazards model was used to calculate hazard ratios (HRs), controlling for potential confounders.

**Results:**

CFS score was negatively correlated with the Barthel index of activities of daily living (Spearman's ρ = −0.83). Of the 729 patients, 253 (35%) were frail. In multivariable analyses adjusting for potential confounders including age and disease stage, frailty was independently associated with shorter RFS (multivariable HR: 1.70, 95% confidence interval: 1.25‐2.31, *P* < .001) and OS (multivariable HR: 2.04, 95% confidence interval: 1.40‐2.99, *P* < .001). There were no significant interactions of frailty with age and disease stage regarding RFS and OS (*P*_interaction_ > .72).

**Conclusion:**

Preoperative frailty was independently associated with shorter RFS and OS following resection of nonmetastatic colorectal cancer, regardless of age and disease stage. Further trials are needed to establish treatment strategies for frail patients with colorectal cancer.

## Graphical abstract

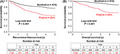

## 1 INTRODUCTION

Colorectal cancer is the most common cancer in Japan and the third most common cancer worldwide.^1^ Approximately 60% of colorectal cancer is diagnosed in patients aged ≥ 65 years, with a median age at diagnosis of 68 years.^2^ Populations around the world are aging rapidly, resulting in the increasing requirement for surgery for colorectal cancer in older patients.^3^ Older patients have age‐related declines in organ function and host immunity.^4^ Because many clinical trials for colorectal cancer did not include patients with colorectal cancer ≥75 years of age,^5^ there is a lack of evidence‐based guidelines for older patients with colorectal cancer.

Frailty is defined as a state of reduced physiological reserve from pathological or iatrogenic stressors because of age‐related impairments.^6^ Frail patients with cancer have been associated with poor treatment tolerance and high incidence of postoperative complications.^7^, ^8^, ^9^ Hence, identifying frail patients with cancer could facilitate improvements in clinical outcomes following surgery. The Clinical Frailty Scale (CFS) is a validated assessment tool that provides a generally accepted clinical definition of frailty, and higher scores are associated with worse clinical outcomes following cardiovascular procedures and surgery.^10^ Three previous studies examined an association of frailty with overall mortality, especially in older patients with colorectal cancer (N < 200 in each study).^11^, ^12^ However, associations of frailty with recurrence and mortality following resection for colorectal cancer in all age groups remain unclear. We hypothesized that preoperative frailty according to the CFS might be associated with worse clinical outcomes following curative resection of nonmetastatic colorectal cancer.

To test this hypothesis, we analyzed data for 729 consecutive patients with stage I–stage III colorectal cancer following curative resection and examined preoperative frailty according to the CFS in relation to recurrence and mortality.

## 2 METHODS

### 2.1 Patients

We analyzed data for patients with stage I, stage II, and stage III colon and rectal carcinoma who underwent curative resection at the National Hospital Organization Kumamoto Medical Center between January 2009 and December 2016. None of the patients with rectal cancer received neoadjuvant chemotherapy or radiotherapy. The main inclusion criteria were as follows: (i) patients aged over 18 years; (ii) histologically confirmed stage I–stage III colorectal adenocarcinoma after curative resection; and (iii) no other active malignancy.

This study was approved by the Human Ethics Review Committee of the National Hospital Organization Kumamoto Medical Centre, Kumamoto, Japan (institutional ethical committee number: 907); the requirement for written informed consent was waived in view of the retrospective nature of the study.

Preoperative frailty was assessed by physicians or trained medical professionals using the CFS according to the Canadian Study of Health and Aging grading criteria.^10^ The CFS ranged from 1 (very fit) to 9 (terminally ill). We considered patients to be frail if they had a score ≥4, according to previous studies.^10^

The functional status of patients at hospital admission was assessed using the Barthel index of activities of daily living (ADLs), which measures the level of functional independence in the following six categories: bathing; dressing; using a bathroom; moving from one place to another; continence; and feeding.^13^ The index yields a score of 0‐100 points, where 100 points signifies full independence.

Recurrence‐free survival (RFS) was defined as the time from surgery to recurrence or death. Overall survival (OS) was calculated as the time from surgery to death from any cause. Distant metastasis was defined as any tumor recurrence in the peritoneum, distant lymph node, or distant organs, including liver and lungs, with or without locoregional recurrence. Locoregional recurrence was defined as any tumor recurrence in the surgical bed, the site of anastomosis, or regional lymph node without distant metastasis. A single institutional pathologist diagnosed the depth of wall invasion, status of lymph node metastasis, and histopathological differentiation based on the Japanese Classification of Colorectal Carcinoma.^14^ Tumor location (cecum, ascending colon, transverse colon, descending colon, sigmoid colon, rectosigmoid colon, and rectum) was recorded based on lower endoscopy, computed tomographic colonography, or operative findings. The proximal colon consisted of the cecum, ascending colon, and transverse colon, whereas the distal colon consisted of the descending colon, sigmoid colon, and rectosigmoid colon. Postoperative complications were recorded and graded as defined by the Clavien–Dindo classification system.^15^ Preoperative blood samples were obtained within 2 weeks of resection for colorectal cancer. Patients’ nutritional status was assessed using the prognostic nutritional status score, which was calculated using admission data as follows: 10 × serum albumin (g/dL) + 0.005 × total lymphocyte count (per mm^3^).^16^ We used the definition of anastomotic leakage as previously reported in clinical trials^17^; peritonitis from any staple line, and pelvic abscess without a radiologically proven leakage mechanism were included. Leakage was verified by clinical (inspection of the drain contents), endoscopic (flexible sigmoidoscopy), or radiologic (rectal contrast study, computed tomography) interventions.

The current study was reported according to the STROBE guidelines.^18^

### 2.2 Statistical analysis

All statistical analyses were performed using JMP (version 12.2, SAS Institute), and all *P* values were two‐sided. All statistical tests were two‐sided at an α level of 0.005, considering multiple comparisons and consequent false positives.^19^

The Kaplan–Meier method was used to describe the RFS and OS distributions with the log‐rank test. The log‐rank test for trend was performed to assess a linear trend in survival probability across the ordinal categories (1‐3 [0], 4, 5 [1], and ≥6 [2]) of the CFS scores. A Cox proportional hazards model was used to compute hazard ratios (HRs) and confidence intervals (CIs). Age‐adjusted HRs for RFS and OS in frail patients were calculated from Cox proportional hazards models that adjusted patient age at surgery (continuous variable). Multivariable Cox proportional hazards regression models were used to identify the independent risk factors for RFS and OS. The multivariable models included variables showing a univariable association (*P* < .05) with RFS or OS. The statistical interaction was assessed by the Wald test on the cross‐product term of frailty (binary categories: nonfrail [0] and frail [1]) with age (binary categories: <75 [0] and ≥75 [1]) and disease stage (ordinal categories: I [1], II [2], and III [3]) variables in a Cox proportional hazards regression model.

Because the CFS score and the Barthel index of ADLs did not fit a normal distribution with the use of the Shapiro–Wilk test for normality (*P* < .0001), Spearman's correlation was used to evaluate the correlation between the CFS score and the Barthel index of ADLs. We performed multivariable logistic regression analysis to assess independent factors for preoperative frailty. A backward stepwise elimination with a threshold of *P* < .05 was used to select variables in the final models.

Categorical variables are presented as proportions. Non‐normally distributed variables were reported as medians with interquartile ranges (25%‐75%). Categorical data were compared using the chi‐square test or Fisher's exact test, and non‐normally distributed data were compared using the Mann–Whitney U test.

## 3 RESULTS

### 3.1 Clinicopathological features and perioperative outcomes according to the CFS score

**FIGURE 1:**
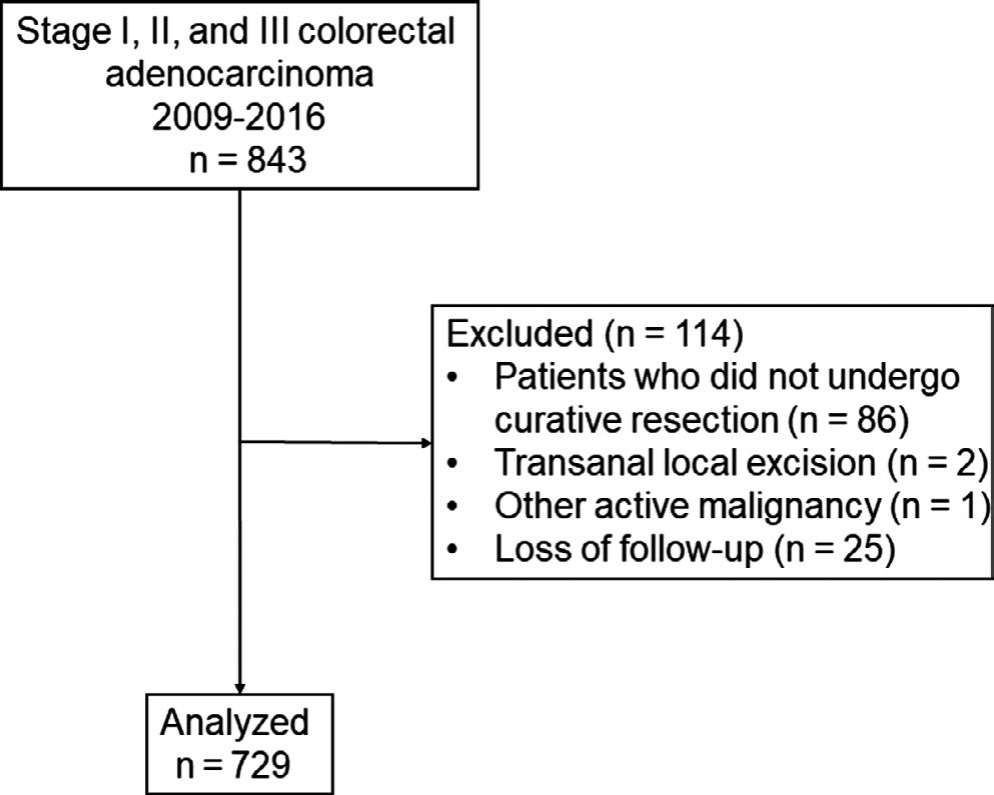
Study flow diagram of patients with stage I, stage II, and stage III colon and rectal carcinoma who underwent curative resection at the National Hospital Organization Kumamoto Medical Center between January 2009 and December 2016

We assessed the preoperative CFS in a total of 729 patients with stage I, stage II, and stage III colorectal adenocarcinoma who underwent curative resection (Figure 1). The CFS score was highly correlated with the Barthel index of ADLs (Spearman's *ρ* = −0.83; *P* < .001).

Of the 729 patients, 253 (35%) were frail and 476 (65%) were nonfrail. Table 1 and Table S1 summarize clinicopathological features and perioperative outcomes according to frailty. Frailty in patients with stage I–stage III colorectal cancer was associated with advanced age, low body mass index, poor American Society of Anesthesiologists physical status classification, proximal colon cancer, high serum carcinoembryonic antigen and carbohydrate antigen 19‐9 levels, advanced disease stage, lower preoperative prognostic nutritional index, large volume of intraoperative bleeding, and absence of adjuvant chemotherapy (all *P* < .005). In multivariable logistic regression analysis, advanced age (*P* < .001), poor American Society of Anesthesiologists physical status classification (*P* = .010), low prognostic nutritional index (*P* < .001), and low Barthel index of ADLs (*P* < .001) were independently associated with preoperative frailty.

**TABLE 1:** Clinical and pathological characteristics according to frailty

Characteristic^a^	All patients (n = 729)	Nonfrail (n = 476)	Frail (n = 253)	*P* value^b^
Gender				
Men	385 (53%)	266 (56%)	119 (47%)	.023
Women	344 (47%)	210 (44%)	134 (53%)	
Age in years				
<75	397 (54%)	307 (65%)	90 (36%)	<.001
≥75	332 (46%)	169 (35%)	163 (64%)	
Body mass index (kg/m^2^)				
<25	580 (80%)	362 (76%)	218 (86%)	.001
≥25	149 (20%)	114 (24%)	35 (14%)	
ASA‐PS				
1 or 2	574 (79%)	413 (87%)	161 (64%)	<.001
3 or 4	155 (21%)	63 (13%)	92 (36%)	
Obstruction or perforation				
Absent	665 (91%)	445 (93%)	220 (87%)	.003
Present	64 (8.8%)	31 (6.5%)	33 (13%)	
Emergency operation				
Absent	698 (96%)	462 (97%)	236 (93%)	.016
Present	31 (4.3%)	14 (2.9%)	17 (6.7%)	
Tumor location				
Proximal colon	269 (37%)	152 (32%)	117 (46%)	<.001
Distal colon	310 (42%)	214 (45%)	96 (38%)	
Rectum	150 (21%)	110 (23%)	40 (16%)	
CEA				
<5 ng/mL	420 (58%)	298 (63%)	122 (48%)	<.001
≥5 ng/mL	309 (42%)	178 (37%)	131 (52%)	
CA19‐9				
<37 U/mL	651 (89%)	437 (92%)	214 (85%)	.003
≥37 U/mL	78 (11%)	39 (8.2%)	39 (15%)	
The Barthel index of ADLs				
0‐59	160 (22%)	14 (2.9%)	146 (58%)	<.001
60‐84	62 (8.5%)	11 (2.3%)	51 (20%)	
85‐100	507 (70%)	451 (95%)	56 (22%)	
Prognostic Nutritional Index				
Median (IQR)	62 (50‐75)	68 (57‐80)	51 (40‐61)	<.001
Disease stage				
I	159 (22%)	122 (26%)	37 (15%)	.002
II	323 (44%)	197 (41%)	126 (50%)	
III	247 (34%)	157 (33%)	90 (35%)	
Tumor differentiation				
Well	683 (94%)	451 (95%)	232 (92%)	.11
Poor or mucinous	46 (6.3%)	25 (5.3%)	21 (8.3%)	

Abbreviations: ASA, The American Society of Anesthesiologists; ASA‐PS, ASA physical status classification; CA19‐9, carbohydrate antigen 19‐9; CEA, carcinoembryonic antigen; IQR, interquartile range.

^a^ Categorical variables are presented as proportions. Non‐normally distributed variables are reported as medians with interquartile ranges.

^b^ Categorical data were compared using the chi‐square test or Fisher's exact test. Non‐normally distributed data were compared using the Mann‐Whitney U test.

Four patients (0.4%) died within 90 days following surgery. Associations between frailty and postoperative outcomes, namely 90‐day mortality, the incidence of anastomotic leakage, and postoperative complications ≥ Clavien–Dindo grade III, were not statistically significant (*P* > .24; Table S1).

### 3.2 Associations of frailty with RFS and OS in stage I, stage II, and stage III colorectal cancer following curative resection

After excluding the four patients who died within 90 days, we examined the associations of frailty with RFS and OS in 725 patients with stage I‐stage III colorectal cancer. Twenty‐six (3.6%) patients had locoregional recurrence, and 115 (16%) patients had distant metastasis. Compared with nonfrail patients, frail patients were more likely to have distant metastasis (13% vs 21%, *P* = .016; Table S2). Frail patients were less likely to receive chemotherapy or surgical resection for recurrent colorectal cancer than nonfrail patients (*P* < .001; Table S2).

**FIGURE 2:**
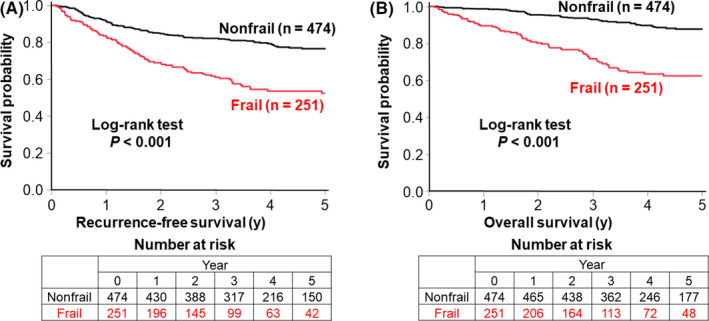
Kaplan–Meier curves for recurrence‐free survival (A) and overall survival (B) according to preoperative frailty. The *P* value was calculated by the log‐rank test (two‐sided)

The median follow‐up was 3.5 years (interquartile range: 2.5‐5.1 years). In the Kaplan–Meier analyses, preoperative frailty was associated with shorter RFS (*P* < .001 by the log‐rank test; Figure 2A) and OS (*P* < .001 by the log‐rank test; Figure 2B). We also used three ordinal categories of the CFS scores (CFS 1‐3 vs 4, 5 vs ≥6) and observed a statistically significant trend for shorter RFS (*P* < .001 by the log‐rank test for trend) and OS (*P* < .001 by the log‐rank test for trend) with an increase in the CFS scores (Figure S1).

We also examined the association of frailty with RFS according to disease stage and age. We observed significant associations of frailty with shorter RFS in stage I (*P* = .024), stage II (*P* < .001), and stage III patients (*P* < .001 by the log‐rank test; Figure S2). Frailty was also associated with shorter RFS in patients aged <75 years (*P* < .001) and ≥75 years (*P* < .001 by the log‐rank test; Figure S3). Compared with nonfrail patients, age‐adjusted HRs (95% confidence interval) for RFS and OS in frail patients were 2.02 (1.52‐2.69) and 3.04 (2.13‐4.35), respectively.

In the multivariable Cox regression analyses after adjusting for potential confounders, namely age, disease stage, and adjuvant chemotherapy, preoperative frailty remained an independent factor, showing significant associations with shorter RFS (*P* < .001; Table 2) and OS (*P* < .001; Table 3). Compared with nonfrail patients, multivariable HRs (95% confidence interval) for RFS and OS in frail patients were 1.70 (1.25‐2.31) and 2.04 (1.40‐2.99), respectively.

**TABLE 2:** Associations of frailty with recurrence‐free survival after curative resection in 725 patients with stage I–stage III colorectal cancer

	Univariable HR (95% CI)	*P*	Multivariable HR (95% CI)^a^	*P*
*Recurrence‐free survival*				
Preoperative frailty				
Frail (vs nonfrail)	2.33 (1.78‐3.05)	<.001	1.70 (1.25‐2.31)	<.001
Age in years				
≥75 (vs <75)	1.77 (1.35‐2.33)	<.001	1.39 (1.02‐1.88)	.034
ASA‐PS				
3 or 4 (vs 1 or 2)	1.51 (1.11‐2.04)	.010	0.93 (0.67‐1.29)	.67
Obstruction or perforation				
Present (vs absent)	1.73 (1.14‐2.53)	.012	1.49 (0.97‐2.23)	.07
Tumor location				
Proximal colon (vs distal colon)	1.46 (1.07‐1.99)	.016	1.23 (0.89‐1.70)	.21
Rectum (vs distal colon)	1.44 (1.00‐2.05)	.049	1.56 (1.05‐2.30)	.027
CEA level				
≥5 ng/mL (vs <5 ng/mL)	1.55 (1.19‐2.04)	.001	0.97 (0.72‐1.31)	.85
CA19‐9 level				
≥37 U/mL (vs <37 U/mL)	2.34 (1.62‐3.29)	<.001	1.86 (1.26‐2.68)	.002
Intraoperative bleeding				
≥200 mL (vs <200 mL)	1.68 (1.28‐2.21)	<.001	1.36 (1.02‐1.80)	.039
Anastomotic leakage				
Present (vs absent)	1.79 (1.11‐2.75)	.019	1.77 (1.05‐2.85)	.032
Disease stage				
II (vs I)	1.41 (0.94‐2.18)	.10	1.27 (0.82‐2.01)	.28
III (vs I)	2.62 (1.78‐3.97)	<.001	2.85 (1.83‐4.53)	<.001
Adjuvant chemotherapy				
Present (vs absent)	0.57 (0.40‐0.79)	<.001	0.45 (0.30‐0.65)	<.001

Abbreviations: ASA, The American Society of Anesthesiologists; ASA‐PS, ASA physical status classification; CA19‐9, carbohydrate antigen 19‐9; CEA, carcinoembryonic antigen; CI, confidence interval; HR, hazard ratio.

^a^ Multivariable Cox proportional hazards regression models included variables showing a univariable association (*P* < .05) with recurrence‐free survival.

**TABLE 3:** Associations of frailty with overall survival after curative resection in 725 patients with stage I–stage III colorectal cancer

	Univariable HR (95% CI)	*P*	Multivariable HR (95% CI)^a^	*P*
*Overall survival*				
Preoperative frailty				
Frail (vs nonfrail)	3.57 (2.54‐5.04)	<.001	2.04 (1.40‐2.99)	<.001
Age in years				
≥75 (vs <75)	2.41 (1.71‐3.42)	<.001	1.60 (1.11‐2.33)	.011
ASA‐PS				
3 or 4 (vs 1 or 2)	2.23 (1.55‐3.16)	<.001	1.21 (0.82‐1.76)	.33
Emergency operation				
Present (vs absent)	2.12 (1.12‐3.64)	.022	1.66 (0.88‐2.89)	.11
CEA level				
≥5 ng/mL (vs <5 ng/mL)	1.65 (1.18‐2.32)	.004	1.12 (0.78‐1.63)	.54
CA19‐9 level				
≥37 U/mL (vs <37 U/mL)	2.67 (1.72‐4.01)	<.001	1.95 (1.23‐3.00)	.005
Intraoperative bleeding				
≥200 mL (vs <200 mL)	1.47 (1.04‐2.07)	.029	1.14 (0.80‐1.63)	0.47
Disease stage				
II (vs I)	1.14 (0.69‐1.94)	.62	0.97 (0.56‐1.70)	0.90
III (vs I)	2.34 (1.48‐3.85)	<.001	2.72 (1.61‐4.74)	<0.001
Adjuvant chemotherapy				
Present (vs absent)	0.26 (0.15‐0.43)	<.001	0.23 (0.12‐0.40)	<0.001

Abbreviations: ASA, The American Society of Anesthesiologists; ASA‐PS, ASA physical status classification; CA19‐9, carbohydrate antigen 19‐9; CEA, carcinoembryonic antigen; CI, confidence interval; HR, hazard ratio.

^a^ Multivariable Cox proportional hazards regression models included variables showing a univariable association (*P* < .05) with overall survival.

We confirmed no significant interactions between frailty and age in the RFS (*P*_interaction_ = .73) and OS (*P*_interaction_ = .72) analyses. We also confirmed no significant interactions between frailty and disease stage in the RFS (*P*_interaction_ = .98) and OS (*P*_interaction_ = .96) analyses.

## 4 DISCUSSION

We performed this study to test the hypothesis that preoperative frailty might be associated with worse clinical outcomes following curative resection of colorectal cancer. We found that preoperative frailty was independently associated with shorter RFS and OS in stage I, stage II, and stage III colorectal carcinoma following curative resection, regardless of age, disease stage, and adjuvant chemotherapy.

Three previous studies examined the prognostic association of frailty in older patients with colorectal cancer. In 143 older patients with stage IV colorectal cancer ≥75 years of age who underwent palliative chemotherapy, frail patients are associated with an increased risk of overall mortality.^20^ Two cohort studies have shown that frail patients are associated with an increased risk of overall mortality following resection in older patients with colorectal cancer ≥70 years of age.^11^, ^12^ To our knowledge, no previous study has shown the association of frailty with recurrence and mortality following curative resection for colorectal carcinoma, after adjusting for key prognostic factors including age and disease stage.

No consensus exists on the most appropriate definition of frailty or the most clinically useful tool to assess frailty. The CFS grading derived by the Canadian Study of Health and Aging committee is one of the most reliable methods to assess frailty, and higher scores have been associated with worse clinical outcomes following cardiovascular procedures and surgery.^10^, ^21^ Although the CFS grading tool is disadvantaged by its semi‐quantitative classification, the current study identified significant correlations between CFS scores and several other indicators of frailty, namely advanced age, low Barthel index of ADLs, and poor nutritional status,^22^ which supports the feasibility and utility of the CFS in patients with colorectal cancer. Although further prospective clinical trials are needed to examine the association between preoperative frailty and patient survival in colorectal cancer, the current study suggests that the assessment of preoperative frailty can be integrated into clinical practice to improve risk assessment in patients with colorectal cancer.

Colorectal cancers are a heterogeneous group of diseases that result from the accumulation of differing sets of genomic and epigenomic alterations, and tumor–host interactions.^23^, ^24^ The mechanisms underlying the associations between frailty and recurrence and mortality following resection of colorectal cancer are poorly understood. Frailty has been associated with high levels of C‐reactive protein and interleukin‐6, suggesting that chronic inflammation may play an important role in the pathogenesis of frailty.^25^ The current study identified significant associations of preoperative frailty with low preoperative nutritional status that has been associated with impaired antitumor immune response and worse prognosis following resection of colorectal cancer.^16^, ^26^ These lines of evidence, together with the findings from the current study, support the hypothesis that frailty may represent a risk factor for recurrence and mortality after resection of colorectal cancer, in part through the systemic inflammatory response and the antitumor immune response, although further studies are needed to clarify the exact mechanism. Frail patients with cancer have been associated with poor tolerance to chemotherapy.^27^ In the current study, frail patients with colorectal cancer were less likely to receive adjuvant chemotherapy, and chemotherapy or surgical resection for recurrent colorectal cancer than nonfrail patients. These findings may explain the associations of frailty with recurrence and mortality following resection of colorectal cancer. Further investigations may be needed to explore potential influences of frailty on outcomes of adjuvant chemotherapy, and chemotherapy or surgical resection for recurrent colorectal cancer.

The management of frail patients with colorectal cancer is challenging. In the current study, frail patients had poor nutrition status and poor ADL scores. Perioperative nutritional support enhances host immunity in patients with gastrointestinal cancers.^28^ Older patients who participate in rehabilitation programs can improve their level of independence as well as decrease their mortality risk after cardiac surgery,^29^ which suggests that the degree of frailty may be reversible. Nutritional support and prehabilitation, including exercise training and promotion of physical activity, significantly decreased length of hospital stay and reduced postoperative complications in patients undergoing major abdominal surgery.^30^ Hence, further investigations may be warranted to explore potential influences of perioperative nutritional support, prehabilitation, or rehabilitation on clinical outcomes in frail patients with colorectal cancer following curative resection.

We acknowledge several limitations in the current study. First, the CFS is semi‐quantitative and subjective in nature, and therefore, it is predisposed to interobserver variability. This potential variability would have driven our results towards the null hypothesis. Despite this limitation, we were able to demonstrate significant and independent associations of preoperative frailty with shorter RFS and OS. The second limitation is the retrospective and single‐center design. Hence, the findings of the current study need to be validated by future nationwide, multicenter, or propensity score‐matched studies. Nonetheless, we believe that our analysis represents a valuable hypothesis‐generating study that can guide future conclusive studies. Third, we did not examine tumor molecular features or immune cells in patients’ colorectal cancer tissues. Thus, further investigations are needed to examine the potential influence of frailty on tumor molecular features and antitumor immunity in colorectal cancer.

A major strength of this study was that it included a large number of older and frail patients, which enabled us to assess the prognostic significance of frailty in colorectal cancer, controlling for the potential confounders of age, disease stage, and adjuvant chemotherapy.

In conclusion, preoperative frailty was independently associated with shorter RFS and OS after curative resection in nonmetastatic colorectal cancer, regardless of age and disease stage. The CFS may be a useful preoperative assessment tool for predicting recurrence and mortality following resection for nonmetastatic colorectal cancer. Further clinical trials are needed to establish a standardized assessment of frailty and treatment strategies for frail patients in colorectal cancer.

## DISCLOSURES

Funding: This study was supported by a grant from JSPS KAKENHI (grant number 17H05094).

Conflict of Interest: The authors declare no conflict of interest.

Author contribution: All authors are in agreement with the content of the manuscript.

## Supporting information

Figure S1

Figure S2

Figure S3

Table S1

Table S2

Supplementary Material
