# Supplementary figures and images for: Frailty is an independent risk factor for recurrence and mortality following curative resection of stage I–III colorectal cancer
Source: Ann Gastroenterol Surg. 2020 Apr 19;4(4):405–12. doi: 10.1002/ags3.12337 (PMC7382441; doi:10.1002/ags3.12337)

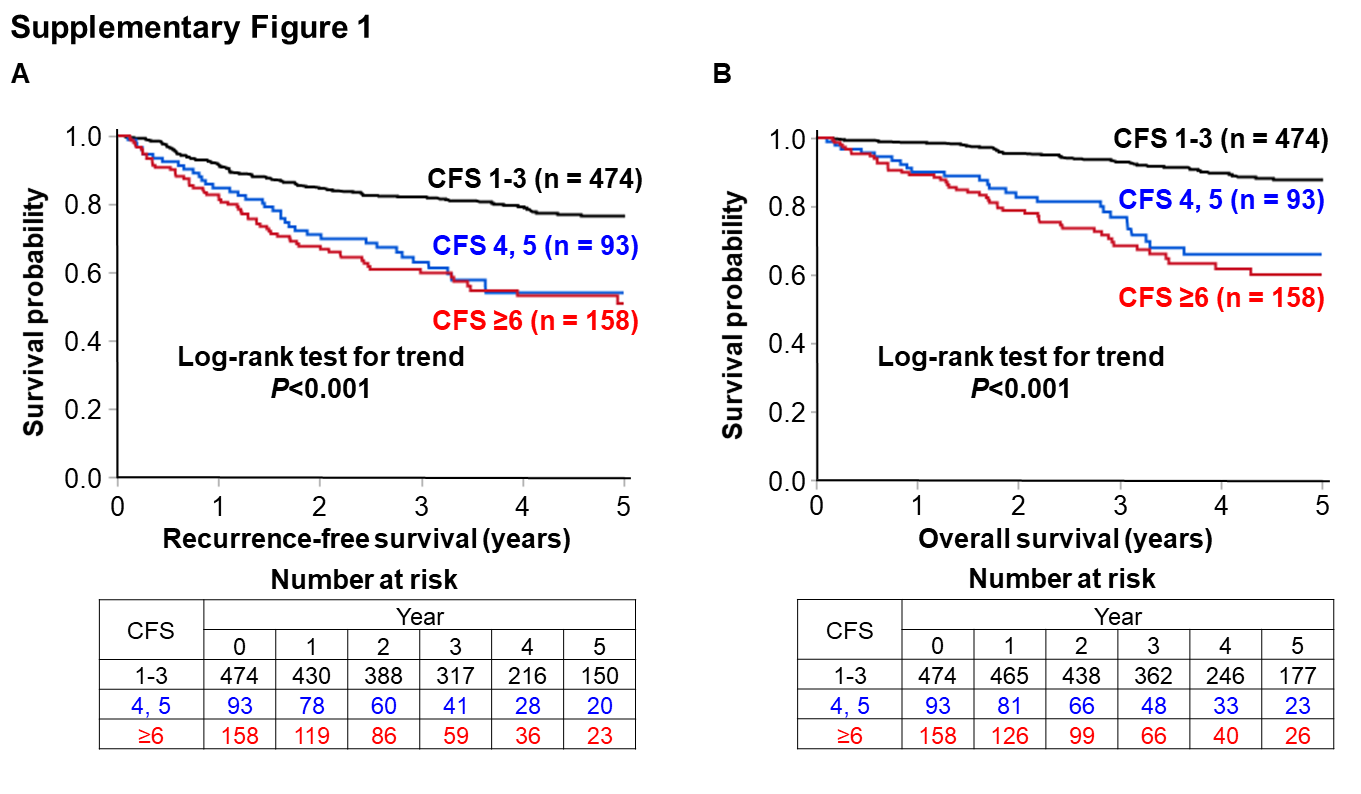

Supplement: Supplementary file 1 — Figure S1 [file AGS3-4-405-s001.tif]

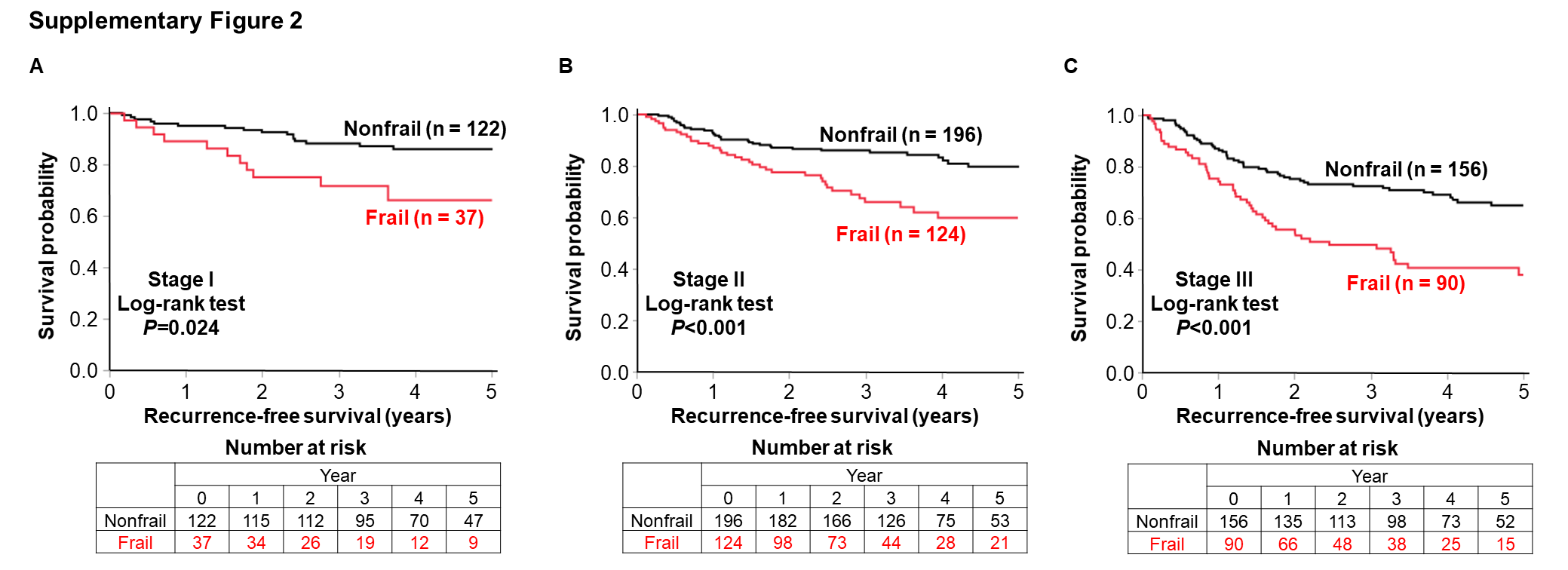

Supplement: Supplementary file 2 — Figure S2 [file AGS3-4-405-s002.tif]

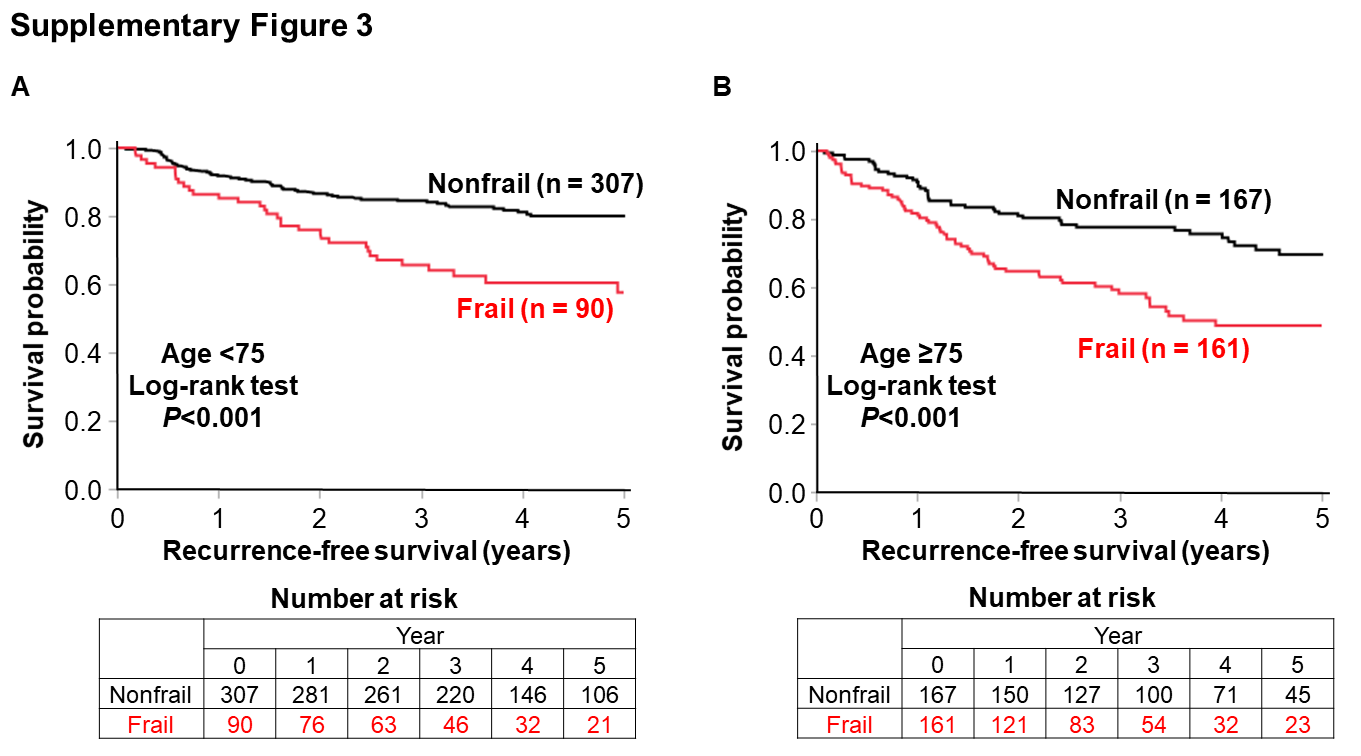

Supplement: Supplementary file 3 — Figure S3 [file AGS3-4-405-s003.tif]
