# Supplementary material for: Frailty is an independent risk factor for recurrence and mortality following curative resection of stage I–III colorectal cancer
Source: Ann Gastroenterol Surg. 2020 Apr 19;4(4):405–12. doi: 10.1002/ags3.12337 (PMC7382441; doi:10.1002/ags3.12337)
Supplement: Supplementary file 4 — Table S1 [file AGS3-4-405-s004.doc]

Supplementary Table 1. Perioperative outcomes according to frailty.

| Characteristic^†^ | All patients  (n = 729) | Nonfrail  (n = 476) | Frail  (n = 253) | *P* value^‡^ |
| --- | --- | --- | --- | --- |
| Intraoperative bleeding (mL) |  |  |  | 0.003 |
| <200 | 509 (70%) | 350 (74%) | 159 (63%) |  |
| ≥200 | 220 (30%) | 126 (26%) | 94 (37%) |  |
| 90-day mortality |  |  |  | 0.52 |
| No | 725 (99%) | 474 (99%) | 251 (99%) |  |
| Yes | 4 (0.6%) | 2 (0.4%) | 2 (0.8%) |  |
| Anastomotic leakage |  |  |  | 0.24 |
| Absent | 684 (94%) | 443 (93%) | 241 (95%) |  |
| Present | 45 (6.2%) | 33 (6.9%) | 12 (4.7%) |  |
| Postoperative complications ≥Grade III Clavien-Dindo classification |  |  |  | 0.67 |
| No | 667 (92%) | 434 (91%) | 233 (92%) |  |
| Yes | 62 (8.5%) | 42 (8.8%) | 20 (7.9%) |  |
| Adjuvant chemotherapy |  |  |  | <0.001 |
| No | 520 (71%) | 307 (64%) | 213 (84%) |  |
| Capecitabine, UFT, or TS-1 | 150 (21%) | 117 (25%) | 33 (13%) |  |
| XELOX or FOLFOX | 59 (8.1%) | 52 (11%) | 7 (2.8%) |  |

UFT, tegafur-uracil; TS-1, tegafur, gimeracil, and oteracil potassium; XELOX, capecitabine and oxaliplatin; FOLFOX, bolus and infused fluorouracil with oxaliplatin. ^†^Categorical variables are presented as proportions. ^‡^Categorical data were compared using the chi-square test or Fisher’s exact test.
