# Supplementary material for: Frailty is an independent risk factor for recurrence and mortality following curative resection of stage I–III colorectal cancer
Source: Ann Gastroenterol Surg. 2020 Apr 19;4(4):405–12. doi: 10.1002/ags3.12337 (PMC7382441; doi:10.1002/ags3.12337)
Supplement: Supplementary file 5 — Table S2 [file AGS3-4-405-s005.doc]

Supplementary Table 2. Patterns of recurrence and management of recurrence according to frailty.

|  | All patients  (n = 725) | Nonfrail  (n = 474) | Frail  (n = 251) | *P* value^¶^ |
| --- | --- | --- | --- | --- |
| **Patterns of recurrence** |  |  |  | 0.016 |
| Absent | 584 (81%) | 393 (83%) | 191 (76%) |  |
| Distant metastasis^†^ | 115 (16%) | 62 (13%) | 53 (21%) |  |
| Locoregional recurrence^‡^ | 26 (3.6%) | 19 (4.0%) | 7 (2.8%) |  |
|  |  |  |  |  |
| **Management of recurrence** |  |  |  | <0.001 |
| Best supportive care | 51 (36%) | 11 (14%) | 40 (67%) |  |
| Capecitabine, UFT, TS-1 | 11 (7.8%) | 6 (7.4%) | 5 (8.3%) |  |
| Oxaliplatin- or irinotecan-based chemotherapy with or without molecularly targeted therapy^§^ | 44 (31%) | 36 (44%) | 8 (13%) |  |
| Surgical resection | 33 (23%) | 26 (32%) | 7 (12%) |  |
| Radiation therapy | 2 (1.4%) | 2 (2.5%) | 0 |  |

^†^Distant metastasis was defined as any tumor recurrence in the peritoneum, distant lymph node, or distant organs, including liver and lungs, with or without locoregional recurrence. ^‡^Locoregional recurrence was defined as any tumor recurrence in the surgical bed, the site of anastomosis, or regional lymph node without distant metastasis. ^§^Molecularly targeted therapy was a monoclonal antibody against VEGFA (bevacizumab) or EGFR (cetuximab or panitumumab). ^¶^Categorical data were compared using the chi-square test or Fisher’s exact test.
