# Supplementary material for: Frailty is an independent risk factor for recurrence and mortality following curative resection of stage I–III colorectal cancer
Source: Ann Gastroenterol Surg. 2020 Apr 19;4(4):405–12. doi: 10.1002/ags3.12337 (PMC7382441; doi:10.1002/ags3.12337)
Supplement: Supplementary file 6 — Supplementary Material [file AGS3-4-405-s006.doc]

**Supplementary Figure Legends**

Supplementary Figure 1. Kaplan–Meier curves for recurrence-free survival (A) and overall survival (B) according to the Clinical Frailty Scale (CFS) scores (1-3 vs. 4, 5 vs. ≥6). The P value was calculated by the log-rank test for trend (two-sided).

Supplementary Figure 2. Kaplan–Meier curves for recurrence-free survival according to preoperative frailty in stage I (A), II (B), and III (C) patients. The *P* value was calculated by the log-rank test (two-sided).

Supplementary Figure 3. Kaplan–Meier curves for recurrence-free survival according to preoperative frailty in patients aged <75 years (A) and ≥75 years (B). The *P* value was calculated by the log-rank test (two-sided).
